# Capturing Aesthetic Experiences With Installation Art: An Empirical Assessment of Emotion, Evaluations, and Mobile Eye Tracking in Olafur Eliasson’s “Baroque, Baroque!”

**DOI:** 10.3389/fpsyg.2018.01255

**Published:** 2018-08-06

**Authors:** Matthew Pelowski, Helmut Leder, Vanessa Mitschke, Eva Specker, Gernot Gerger, Pablo P. L. Tinio, Elena Vaporova, Till Bieg, Agnes Husslein-Arco

**Affiliations:** ^1^Department of Basic Psychological Research and Research Methods, Faculty of Psychology, University of Vienna, Vienna, Austria; ^2^Department of General Psychology II, University of Würzburg, Würzburg, Germany; ^3^Department of Psychology, Webster Vienna Private University, Vienna, Austria; ^4^Department of Educational Foundations, Montclair State University, Montclair, NJ, United States; ^5^Center for Technology Experience, AIT Austrian Institute of Technology, Vienna, Austria; ^6^Museum Belvedere, Vienna, Austria

**Keywords:** museum study, aesthetic emotion, mobile eye-tracking, installation art, art perception

## Abstract

Installation art is one of the most important and provocative developments in the visual arts during the last half century and has become a key focus of artists and of contemporary museums. It is also seen as particularly challenging or even disliked by many viewers, and—due to its unique *in situ*, immersive setting—is equally regarded as difficult or even beyond the grasp of present methods in empirical aesthetic psychology. In this paper, we introduce an exploratory study with installation art, utilizing a collection of techniques to capture the eclectic, the embodied, and often the emotionally-charged viewing experience. We present results from an investigation of two pieces, both part of Olafur Eliasson’s exhibition “Baroque, Baroque” held at the Belvedere museum in Vienna. These were assessed by pre- and post-viewing questionnaires focusing on emotion, meaning-making, and appraisals, in tandem with mobile eye tracking to consider viewers’ attention to both installed artworks and/or to the museum environment. The data showed differences in participants’ emotional states, appraisals, and visual exploration, which together paint a picture of the aesthetic reactions to the works. These differences also showed how viewers’ appraisal strategies, meaning making, and physical actions facilitated relatively more or less deep engagement with, and enjoyment of, the art. The results are discussed in terms of their implications for museum studies, art education, and theory in empirical aesthetics.

## Introduction

Installation art represents one of the most important, and one of the more empirically vexing, developments in the last 50 years of art’s production and scholarship (Osborne, 2002; Bishop, 2005). Involving a monumental shift in emphasis from traditional bounded sculptures or two-dimensional images placed before a viewer, installation artworks are instead comprised of site-specific pieces that *envelop* an individual, often incorporating aspects of the existing environment and designed to bring about complex sensory and emotional experiences (MOCA, 2002). As such, installation artworks can be particularly intriguing for perceivers who are confronted with a visitor-centered medium that often requires their active and mobile interaction (Reiss, 2001). Art installations are also a key emphasis for contemporary museums, where they have become centerpieces of new collections and monetary investment (Bishop, 2005). For the empirical researcher or art-interested psychologist, installation artworks are also particularly intriguing. They specifically possess features aligning with recent interest in art’s ability to emotionally move and to conceptually challenge the viewer (Pelowski et al., 2017b), as well as raising importance of context (e.g., laboratory vs. gallery) or *in situ* ecologically valid conditions as a major component in the art experience (Pelowski et al., 2017a).

At the same time, despite this interest, installation art also poses particular challenges to its understanding and to its assessment, rooted in the very same aspects of the medium and the responses that it may elicit. Installation pieces often discourage traditional appraisals that involve some type of hedonic judgment (“it is a beautiful or pleasing object”) or mimetic identification (“it is a picture of ‘X”’). Instead, they require the visitor’s consideration of the juxtaposition of space and artwork elements, and often their reflection on emotions, bodily sensations, as well as on ambiguity or confusion in such responses. These aspects are argued to be central to the artworks and perhaps key to their enjoyment. Particularly for the viewer with limited art knowledge, these experiences—as with other concept driven art—can lead to displeasure (Silvia, 2013), and are even given as a major reason for why individuals may not visit contemporary museums or enjoy contemporary art (Eaton and Moore, 2002; Goldie and Schellekens, 2009). This also poses a conundrum for museum directors—as well as for artists—with better understanding of how and why individuals do react to installation pieces of key importance for pragmatic, educational, and curatorial decisions. It is equally important to know how certain responses might lead to more or less appreciation, or even the range of responses that viewers might have to such art. For researchers who would do empirical studies, however, and thus who might begin to answer these pragmatic and scientific questions, because it cannot be collapsed into a static image with a seated viewer, and involves a temporally extended, complex, site–specific interaction, installation art has to-date almost never been empirically considered (Minissale, 2012; Weingarden, 2014; Kranjec, 2015).

In this paper, we report on a first exploratory study designed to begin capturing and partially differentiating the viewer experience of museum-based installation art. This was conducted at the exhibition, *Baroque, Baroque*, by the artist Olafur Elliasson, at the Belvedere Museum in Vienna, Austria, and built around a unique opportunity to collaborate with curators and staff of the museum. By selecting two distinct rooms within the exhibition, and employing a combination of behavioral and eye-tracking methods, we examined the interplay amongst viewers’ emotions, artwork evaluations, meaning interpretations, and perception or attention patterns as they engaged with the art. Although designed from the output to be an exploratory test case, this study, through its unique blending of approaches, is hoped to provide both a range of means and supporting evidence for future work, unlocking the unique experiences and key engagement features with these complex and intriguing artworks. Below, we also briefly review the main issues and the unique qualities of installation art as well as past studies, in tandem with a handful of research questions, which we feel give a good starting point for considering some of the most salient issues and our selected methods of analysis. We conclude with a brief discussion of implications and ideas for future research.

## Review—Installation Art: Research Questions, Issues, and Approaches

Installation artworks might in fact be said to present a particularly perfect intersection of issues at the forefront of suggested new directions in empirical/psychological aesthetics. Beginning with the work of Alan Kaprow (e.g., 1950s; see Martinique, 2016) and becoming more common throughout the 1970s and to today, this medium again is defined by a process whereby an artist takes an existing space, often in public areas, in nature, or a room/gallery, and designs the entire environment to create conditions for a cohesive, unique, interactive experience. This can of course be done in any number of ways with different foci or materials. The earliest Kaprow works literally filled a room with various objects such as old tires, or sheets of plastic, colored cloth, etc. often paired with electronic sounds or music. Christo and Jeanne-Claude famously created numerous outdoor installations by for example wrapping the Berlin Reichstag in cloth or placing cloth gates throughout New York’s Central Park through which one might pass while walking (Martinique, 2016). Other artists such as Bruce Nauman or James Turrell use colored lights or words in gallery spaces to confront or effect the mood of a viewer. Ai Wei Wei recently filled museum rooms with millions of porcelain sunflower seeds. Damien Hirst has installed glass cases with bisected animals; and the artist of the present study, Olafur Eliasson, converted a gallery in Denmark to reproduce a riverbed in his native Iceland or filled an entrance hall at London’s Tate Modern with a looming, setting sun.

Such artworks might lead to multiple ways of engaging and participant responses, much as with other art media. However, certain factors are potentially most prominent and thus key candidates to empirically consider: First, installations tend to cause and/or emphasize the foregrounding of some aspect of the viewer’s affective responses, especially felt emotion. This has to do with the designed enveloping nature of the medium, which coincides with an expectation for the presence of a perceiver who, by engaging within the space and interacting with all senses, in a way completes the artwork. Viewers may have a basic awareness and appreciation or remembrance of certain sensations—such as the warmth and intensity or even melancholy of squinting and staring into a sun or walking in an icy barren land (Eliasson). They may feel the weight and leaning sensations from huge blocks of metal mirrored in their own body (Richard Serra; see Kapoula et al., 2011). Bruce Nauman’s Green Light Corridor is known for negative responses, evoking a feeling of discomfort, disgust or claustrophobia (Griswold et al., 2013). Installation artists might in fact often be said to design their works specifically to evoke specific reactions, considering how their environments will impact the viewer’s mood and body. The “participatory” nature of this medium (Novitz, 2001; see also Griswold et al., 2013) may in turn directly require some active awareness of emotions by a viewer and reflection on the nature and importance of their affective experience, and which may also be one artistic *point* of installations.

By creating an encompassing space often with a moving viewer, installation artworks may also act to “walk an individual through” an evolving encounter, and individuals might experience several emotions or evaluations within one experience. For example, a space may be designed to slowly reveal new features. Artworks may also require some acclimation or dawning awareness of different senses. Artists may also anticipate juxtapositions which could lead to mixed (positive and negative) or changed response. One might feel an initial discomfort or oppression, say an overly bright light as in Eliasson, or disgust from being confronted by an embalmed animal (Hirst), followed by relief, interest, or aesthetic appreciation.

This also raises another key aspect, regarding some amount of incongruity, insight, learning, or changed understanding. By foregrounding certain features, installations may serve to force an awareness or deeper consideration. An artist may cause an individual to consider how responses are typically taken for granted, or individuals may even come to deeper appreciation by juxtaposing visual and proprioceptive sensations (awareness of being in a gallery). For example, as noted by Sherman and Morrissey (2017), the works of James Turrell—denoted by rooms flooded with differently colored light—have been “described… as transformative. The immersive light environments cause one’s own perception to become the object of reflection and led many to a deeper understanding of themselves and their relationship to the external world, whose access to the world is mediated by visual perceptual faculty with particular features, limitations, and abilities.” This may also directly involve a sense of discrepancy between the “art” and the pre-existing environment. By framing certain artistic interventions within, say, a gallery setting, artists may cause individuals to rethink their own responses, to consider different viewpoints (i.e., “aesthetic” vs. more descriptive or every-day evaluations) or even create some confusion regarding what is art or the environment. Installations may also utilize a more overtly cognitive interpretations or meaning-making. The *Sunflower Seeds* by Ai Wei Wei, in addition to presenting a visually compelling stimulus, was also handmade by 1600 artisans and had the goal of commenting on mass production and consumption in conjunction with aesthetic experience (Martinique, 2016). The embalmed animals by Damien Hirst may confront viewers with their own mortality or cause them to question their aestheticizing of death (see Pelowski et al., 2012 for other examples).

These processes, as they relate to meaning interpretations are also argued to be a key element for the appreciation of installation art—suggesting a movement away from more traditional responses or attempts to identify the mimetic content or provenance of a work (who is it by, what is depicted, what is it made of) to a meaning relating the artwork significance to its ability to create the conditions for a reflective, insightful, or emotional experience. Such an “experience-based” meaning, although of course not unique to installation examples (e.g., see Pelowski and Akiba, 2011 for discussion with mimetic or abstract paintings), are an increasingly key aspect of contemporary conceptual and postmodern art (Bourdieu, 1979/1984; Goldie and Schellekens, 2009), and in fact have been argued to represent a more general goal of art viewing or art education, representing a personalized, deepened art experience (e.g., Dewey, 1980; Tucker, 2004; Pelowski and Akiba, 2011).

It is also such features, in conjunction with cognition, reflection, and body/affective responses, that may pose particular difficulty with viewers, who may expect to be faced with requirements for formal appraisals or understanding of content, and may thus not be prepared for an insightful, introspective, or shifting experience. It may also be that certain responses may not coincide with all art examples—for example Christo argued that his works should not have deeper meanings beyond the sensory experience (Martinique, 2016). These may of course differ between viewers, with only certain responses leading to more or less enjoyable, engaged, or meaningful responses.

All of these aspects—diverse emotions, awareness and reflection, meaning making, as well as a tendency to juxtapose environment/expectations and even insight—mark compelling research questions. They also mark the present limitations in empirical approaches. This can be traced in both study approaches as well as the theoretical models for processing art. A recent review of main theoretical bases for assessing art (Pelowski et al., 2016) specifically noted their lack of focus on the above features. Primarily this is due to a theoretical basis in prior models of basic vision and attention to low-level artwork features (color, line, form, content, etc., see e.g., Leder et al., 2004), which tend to then only consider responses to these aspects in art processing experience. This in turn connects to empirical studies, which also focus on basic (positive/negative) appraisals or responses, often with less than 5 s viewing durations (Pelowski et al., 2017a for review). This tends to omit changes or evolutions in art experience.

The above limitations also relate specifically to assessing emotion. While models and studies do often include affect, as well as understanding and appraisals, as major components, these are considered only in the context of basic hedonic pleasure/displeasure, arousal, or visual interest. There are presently few attempts in empirical study to take a simultaneously more detailed and broad perspective. Especially, researchers argue for needed attempts to map and record complex, shifting, and negative varieties (e.g., Silvia, 2009), as well as intense aesthetic responses such as being moved, transformed, or feeling resonance with art. Similar arguments are made for needed analyses of mixed or conflicting emotions (Hosoya et al., 2017). This is also important for insight, discrepancy, or even more top-down reflection, and meaning making, which are also obscured in current models and studies (Pelowski et al., 2017b), which also tend to focus on basic identification or understanding of artwork content, and thus do not consider the more personalized reactions described above. In turn, researchers have argued for the need of a broader focus on these different factors, capturing aesthetic responses arising from the interplay of emotion, cognition, expectations, more open–ended interactions (Tallis, 2008; Brown and Dissanayake, 2009; Augustin et al., 2012; Cross and Ticini, 2012).

Installation art also of course raises empirical issues relating to setting. Unlike many other art examples, installations require a site-specific viewing. This is in contrast to most empirical assessments which typically move the viewer to a laboratory and a computer screen for purposes of control and experimental focus (Cela-Conde et al., 2011; Pelowski et al., 2017a). However, this itself might lead to several issues. From the founding of psychology of art as a scientific field (e.g., Fechner, 1876), scholars have agreed that a major component of art’s impact and character involves the tangible, immediate, and “real” experience of original artworks (Tschacher et al., 2012). Scholars also argue that current experimental approaches, dominated by laboratory studies, may lead to differing types of reactions or may not be able to elicit the emotions and juxtapositions found especially with *in situ* art (Brown and Dissanayake, 2009).

Presently, there are very few studies even of art within galleries or museums (e.g., Pelowski et al., 2017a for review). These primarily have focused only on general appraisals such as liking or beauty, asking participants to both assess a succession of artworks (paintings or photographs) on a monitor and/or also to assess the same artworks within a gallery. Results have in turn shown higher ability of real examples to evoke liking and interest (Locher et al., 2001), longer durations of viewing (Brieber et al., 2014), as well as arousal (e.g., Brieber et al., 2015 which also included sculptures as well as paintings) or emotional intensity (Specker et al., 2017). Certainly, this issue is not unique to even visual art (see also Scherer and Coutinho, 2013 for a similar argument with music), but, in conjunction with the general focus on evoked emotion and body responses, may be particularly salient in installation art study.

The need for more on site, in depth studies has only grown with the increasing awareness of the importance of context in aesthetic judgments. Researchers are becoming concerned with the need to consider art reception as a complex interplay of the expectations of the viewer, the characteristics of the art object, and the conditions in which it is experienced. Installation art also adds the unique aspect of the mobile viewer and the inclusive environment, with an individual who may potentially engage with any aspect of the spaces as part of their encounter. Here too, although some emerging studies have shown the ability to generally track patterns of movement or body responses in the gallery (i.e., heartrate and standing patterns when looking at more or less interesting art, Tröndle et al., 2014a) this has generally not been considered in conjunction with complex emotional/cognitive experience (see Mitschke et al., 2017 for discussion of the key intrigue and need for such studies in empirical aesthetics). Although not specifically necessary, researchers would also benefit from access to curators and artists, especially when considering their making decisions and specific goals for evoking certain response, which may also be useful in forming hypotheses and assessing the efficacy of the works.

We are aware of only two examples of empirical studies that have used installation artworks: Tröndle et al. (2014b) considered reactions to a conceptual installation (“A Label Level, 2009” by Nedko Solakov), composed of black marker comments written on the museum walls and commenting on the gallery’s other displayed paintings, drawings, and sculptures. In this case, the researcher, however, were interested in whether participants would find the installed writing to be an “artwork,” finding a correlation between positive answers and higher ratings for importance, beauty, artistic skill, composition, and even curatorial quality, as well as interpersonal differences in regards to art classification, and frequency of museum visits, expectations that the exhibition would be thought-provoking, would touch all senses, and would have famous artworks, and with general appreciation of other video, performance and installation art. Kapoula et al. (2011) also report intriguing evidence from a study of Richard Serra towers installed in a gallery. Although mentioned only briefly as a pilot assessment within the context of a different study, they fitted viewers with a belt accelerometer while they walked through the installation and found that individuals unconsciously adopted the same degree of lean with their own bodies. These studies are compelling in highlighting unique responses and in suggesting that Installations can routinely be assessed even with lay museum viewers, but again only tangentially touch the most compelling aspects of the medium and the resulting experience.

### An Integrative Approach for a Pilot Study of Installation Art Experience

To begin our assessment, it was thus necessary to combine several approaches and range of assessments which have been employed in our, and others’, previous gallery studies, involving assessments of the more descriptive/global assessment of art experience. This also involved our decision to focus on a handful of features—emotion, aspects of meaning making, general appraisal, and basic patterns of looking—which we felt to be most salient from the above review, and a good beginning point for this analysis. The study utilized a paradigm developed in our previous work in museums, in which participants are asked to interact with works of art, individually contained within one room, and with minimum prompting or intervention. This is then followed by a post-viewing survey.

The primary means of assessment involved soliciting participant reports of their felt emotions using a list of terms meant to capture a range of aspects or outcomes. This followed previous work in appraisal theory (e.g., Silvia, 2005a; Scherer et al., 2006) that suggests that specific emotions might be tied to the personal relationship between viewer and art and through mapping their general incidence may provide a broad qualitative sketch of personal experience. In turn, use of a list of emotions has been recently employed in art studies or with other aesthetic media, often collecting the range of responses felt in one’s total museum interaction or hypothetically related to aesthetic media (e.g., Zentner et al., 2008 with music; Hosoya et al., 2017; Schindler et al., 2017 for general aesthetic responses), but also utilized in specific interactions with particular works of art (Specker et al., 2017).

The emotions employed here were based on a list produced as part of an art processing model (Pelowski and Akiba, 2011; updated in Pelowski et al., 2017b) which did attempt to lay out general main outcomes of art experience. This included a range of responses differentiating between largely facile/unemotional, negative/discrepant or highly positive response, as well as terms recently connected to installation or emotionally resonant art—insight, transformation, joy, thrill, being moved etc. This was used in a previous gallery study (Pelowski et al., 2012; Pelowski, 2015), which approached an installation setting—the Rothko rooms, consisting of series of paintings and individual spaces designed by or highly controlled by the artist—showing ability to meaningfully distinguish between broad varieties of experience and especially to identify particularly resonant or insightful encounters. In order to more fully paint a picture of viewers’ personal/emotional combinations, we also utilized a network modeling technique (e.g., Epskamp and Fried, 2016; Hosoya et al., 2017 for use with art). This method can visually and mathematically group and connect individual emotion responses via their partial correlations, and thus visualizes the strength and interconnections in an emotional space. It is especially useful for descriptive/exploratory studies, clustering items without an underlying assumption of higher-order factors, providing information regarding the centrality, interconnections, and specific importance of items in defining the global affective experience.

The above emotions were also combined with a list of artwork appraisals and also with an assessment of meaning using the open-ended question, “What did the artwork mean?” This has been shown to be effective in augmenting emotional and evaluative scales (see Pelowski et al., 2012; Pelowski, 2015). Specifically, answers can be divided into three general types that can further be used to demarcate the experience: (1) descriptive statements about the formal, historical, or semantic content of an artwork (e.g., it is “a picture of X,” or it is “a painting by Y”); (2) statements regarding an artwork’s lack of meaning; and (3) statements in which the meaning or interpretation of an artwork is related to personal experience, emotions, or bodily sensations (e.g., “it reminded me of Y,” or “it made me feel X”). This allowed us to both consider the general approach of viewers—regarding focus on content/semantic information or emotion—in their interpretations of the artworks’ meaning and significance, as well as to identify particular responses described in the above review.

The typical strategy, used by especially naïve art viewers when assessing paintings or sculptures, again often involves responding with descriptive/semantic interpretations (e.g., focus on the content, materials, physical spaces, or places, etc., Leder et al., 2014). In past studies (Pelowski et al., 2012), more direct descriptive responses have also been shown to coincide with approaches typically taken by (often novice) viewers, and, for the present artworks, are also those responses argued to perhaps not be suitable for installation art. Similarly, finding no meaning or feeling a work to be meaningless often coincides with generally negative emotional and cognitive experience. On the other hand, the latter so-called “experience based” interpretation (e.g., see Pelowski and Akiba, 2011)—which can often include both basic recognition of emotional/cognitive responses as well as insight or even metaphorical meaning assumed to be intended by the artist—was argued to be vital for appreciating installation art. Such interpretations have also been shown, in previous museum studies with abstract or more conceptual pieces (e.g., Pelowski, 2015), to correlate with more positive and rewarding assessments.

By combining these measures, we assessed the following research questions: (1) Do installation artworks produce distinct emotional, evaluative, perceptual, and meaning responses that may be captured and that may differ within-subjects? In addition, (2) How do viewers generally respond to the art, and particularly are specific factors linked to more understanding and enjoyment? More specifically in reference to this second question, we considered if certain emotional responses, or general magnitude of emotion, might lead to more positive appraisals. This was in keeping with the above-argued importance of evoked emotion. We also assessed if similar patterns might be found between experience-based interpretations and/or focus on affective experience in meaning, rather than identifying semantic content, and relatively more enjoyment. (3) We also combined the above behavioral measures with use of mobile eye-tracking in the museum installation space. This approach is only emerging in use with art viewing (Heidenreich and Turano, 2011; Walker et al., 2017). However, the technique, which can record looking patterns of a mobile, walking viewer, is especially useful for an installation environment where any part of the environment might be a point of attention. This study followed our recent work in real–world art interaction contexts, specifically Mitschke et al. (2017) which employed the same technology as viewers walked along a riverside path with a number of installed sculptures, graffiti art, in conjunction with the ambient visual environment. This study showed good ability to record general patterns of looking and attention on the different objects.

Due to lack of previous study and specific hypotheses, we were primarily interested in assessing general areas of attention (by placing attention on eye fixations), and considering whether viewers appreciated the installed elements as stand-alone artworks or as components that interact with the ambient environment. This latter approach was hypothesized to be an important aspect of installation artwork appreciation. We also considered how visual exploration itself (time spent looking at specific areas) relates to the experience of installation pieces. Time of total fixations is considered an important feature of psychological experience, representing attention and salience (Tatler, 2007), and has been shown to positively correlate with the appreciation of art (Brieber et al., 2014; Leder et al., 2016). We expected that longer fixation times on the artworks (as opposed to other elements of the rooms such as floors and ceilings) would coincide with more positive or emotional experiences. At the same time, because other aspects of the rooms might play a role in the experience, this technique allowed us to assess whether participants were assessing these features and how attention to these might also coincide with certain appraisals or outputs. By using a matched group of individuals with and without the glasses, we could also offer one more assessment of the efficacy of this technique itself in the art gallery.

## Materials and Methods

### Stimuli

**FIGURE 1:**
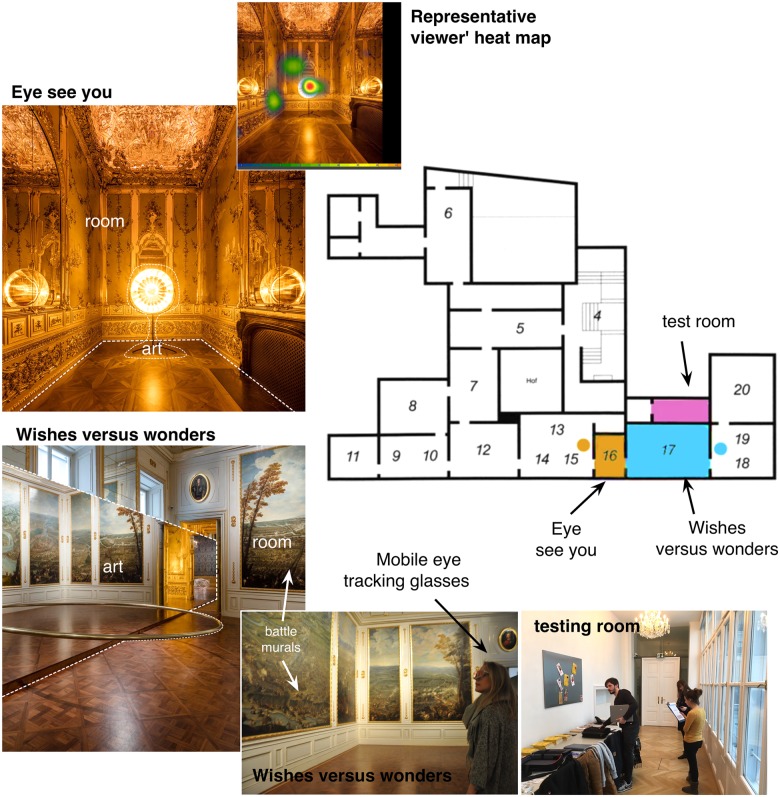
Two installation art rooms, exhibition layout, and details regarding the testing procedure (Note, written informed consents was obtained from all depicted individuals for the publication of the images).

The setting for our study came about through a unique opportunity to collaborate with the staff, artist, and curators at the exhibition, *Baroque, Baroque*, by the artist Olafur Elliasson (November 2015–March 2016), at the Belvedere Museum in Vienna, Austria.^1^ The exhibition consisted of a series of rooms, each containing one installation. According to its curators (Zyman and Codognato, 2015), the exhibition had the purpose of “establishing a dialog between” the baroque architecture and the artworks, creating “surprising affinities between Eliasson’s works and their temporary settings” or exploring “the relation between object and viewer” (Belvedere and Thyssen Bornemisza Art Contemporary, 2015, p. 2). We focused on two pieces (**Figure 1**), which themselves provided two potentially distinct viewing experiences while also combining, potentially, the above emotional and cognitive aspects. The first was *Eye See You* (Eliasson, 2006), a relatively small room (∼3 × 4 m) that contained a sun-like sculpture made from a prefabricated mirror-polished solar cooker and a sodium lamp mounted at its center, which flooded the space with golden light. Two additional dichromatic glass disks changed colors according to a viewer’s position and movement. The installation provided a sense of staring into a warm sun, with a slightly shimmering “moiré effect” (Eliasson, 2017), suggested by the artist and critics to potentially elicit positive and/or melancholic emotion. The second installation, *Wishes* vs. *Wonders* (Eliasson, 2015), was located in a slightly larger room (∼10 × 5 m) and containing a number of painted panels on all walls (original to the building) depicting landscapes and battles. The artist subdivided the room with a tall mirror on which a metal half ring (5 m in diameter) was embedded and which hovered above the parquet floor. According to critics, this installation was expected to provide a “perception of dizzying completeness^2^” or “produc(e) a virtual ring that appeared to float or pass from the actual space…, uncannily traversing the surface of the mirror image” (Husslein-Arco and Habsburg, 2015), but also potentially calling to mind cognitive aspects of war or the human condition as reflected in the design.

### Participants

The study involved 51 participants (all female, *M*_age_ = 22.7 years, *SD*_age_ = 4.7, range: 18–49) who viewed both rooms in a counterbalanced order. Participants were psychology students from the University of Vienna. They had normal or corrected-to-normal vision and participated for course credit. All participants were novice art viewers with no previous training in art making or art history (assessed via a post-test survey). None of the participants had previously seen the installations. The sample was divided into two groups: those who used the eye-tracking glasses (*N* = 24) and those who did not (*N* = 26).

### Procedure

The procedure again followed that reported in our past museum studies (Pelowski, 2015). Participants were met at the entrance of the exhibition hall in groups of two (after having previously signed up for the study and given a scheduled time to make their visit). They were guided to a small room, which was separated from the galleries but still located within the museum. They were asked to provide informed consent and complete a pre-viewing questionnaire (see below). The participants were then separated and one individual from each pair was equipped with eye-tracking glasses that were calibrated (see below, following procedure of our previous art-viewing study by Mitschke et al., 2017). This participant was allowed to briefly acclimate to the use of the glasses. Before starting the gaze recordings, glasses-equipped participants were informed that their gaze behavior and audio were being recorded. Before viewing, participants also completed a series of Likert-type questions assessing their attitudes about art and past art knowledge or experience (7-point, 1 = “Does not apply to me at all”; 7 = “applies absolutely”). This was used as a means of ensuring their novice status.

Each participant was then escorted by a researcher to one of the two rooms containing the installation artworks, which were visually separated (**Figure 1**). Before entering, the participants were instructed as follows: “Please enter this room and stay for as long as you like. You have no time constraints or task requirements. When you are done viewing, please come back to me” (translated from German). After returning from the installation rooms, participants were guided to the testing room and given a post viewing survey about their experience with the specific artwork. The same procedure was then repeated for the second room/installation piece. The amount of time that each participant spent inside each room was recorded. All participants were debriefed upon completion of the study. Participants in the eye-tracking group used the glasses for both rooms, with recalibration done following the first room.

Note also that the inclusion of a glasses and no-glasses equipped group was done both in order to provide a matched control for assessment of potential modulation by wearing glasses themselves, and also for pragmatic reasons, due to museum-imposed limitations on total time for testing. Based on our previous use of this technology (Mitschke et al., 2017) we did not expect any issues to arise from the glasses, and thus it was expected that both glasses and no-glasses groups could be combined in the other analyses.

### Behavioral Self-Report Measures

To assess overall aesthetic experience, we used Likert-type questions related to the following dimensions: Once again, as a main means of assessment, after viewing each room, participants reported incidence and relative magnitude of 20 emotions (see **Figure 5** for all items), based on a similar list and procedure used in a museum study by Pelowski (2015). This has been shown to provide a good range of responses and ability to generally describe the global experience, as well as to meaningfully distinguish between broad varieties, differentiating between largely positive/negative and more or less profound/facile response (see also Ekman et al., 1980; Carstensen et al., 2000 for use of similar lists and Likert-type scale measures in non-art contexts). The list asked participants to report on their emotions via 9-point scale [i.e., “while I was inside the room, I experienced (emotion)”; 0 = “not at all” to 8 = “extremely”].

**FIGURE 2:**
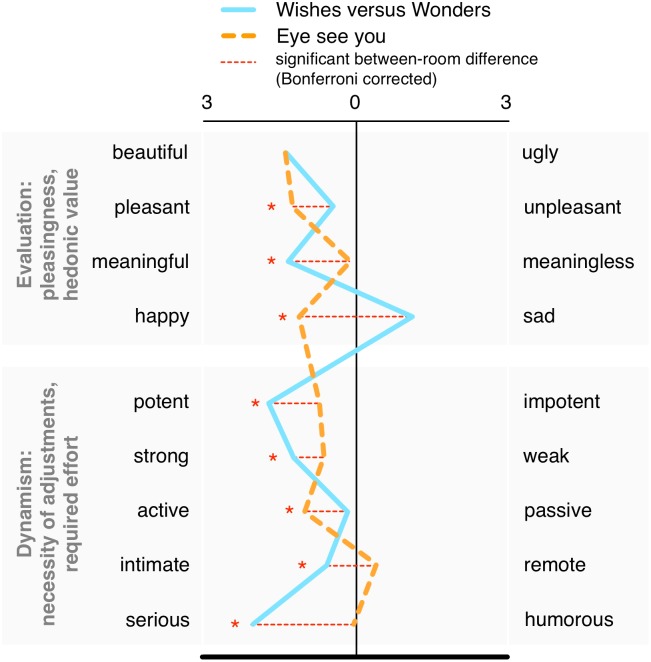
Appraisals (post-viewing) for two rooms of installation art. ^∗^ and dotted line indicate significance at *p* < 0.05, Bonferroni corrected (see also legend above).

Participants then rated the artwork using a series of nine bipolar scales (7-point scale, e.g., 1 = “extremely ugly” – 7 = “extremely beautiful,” all items listed in **Figure 2**). The items, based on the semantic differential technique (Osgood et al., 1957), were selected from a previous museum study (Pelowski, 2015) and combined both hedonic assessments (e.g., liking, pleasing, beauty) as well as terms relating to underlying stimulus potency (strong-weak) and activity (intimate-remote). These latter items—which can also be combined into a more general “dynamism” dimension especially in ratings with art (Kumata and Schramm, 1956; Berlyne, 1974)—have been argued to indicate a relative need for effort/adjustments and thus in conjunction with the above hedonic ratings a relative measure of engagement and/or processing difficulty (Carroll, 1959; see also Berlyne, 1974). In addition, participant’s answers to the hedonic scale “pleasing-displeasing” were expected to be used as a general assessment of art preference or enjoyment (e.g., Silvia, 2005b).

Following this, participant understanding of artwork meaning was assessed using the open-ended question, “What did the artwork mean?” and with the expectation of coding into the three levels of descriptive, no-meaning/no-understanding, and experience-based or emotion/introspection-centered interpretations (following Pelowski et al., 2012; Pelowski, 2015, described in the introduction above).^3^

### Mobile Eye-Tracking

Participants in the eye-tracking group had their fixations and fixation durations measured using a pair of lightweight mobile eye-tracking glasses (iViewETG; SensoMotoric Instruments, Teltow, Germany) that was connected to a small data recording unit (approximately the size of a cellular phone) carried inside a bag worn around the waist and that stored data at a rate of 60 Hz (monocular). The glasses were calibrated (prior to the viewing of each room) inside the testing room with a three-point calibration method using the provided software (following Mitschke et al., 2017).^4^

### Ethics Statement

The study was approved by the ethics committee of the University of Vienna and by the Belvedere Museum. All participants gave informed written consent.

## Results

The results are reported in two sections that are aligned with the research questions: (1) We begin with a general description of the art experience in both rooms that considered viewers’ evaluations, emotion, meaning-making, eye fixations, and between-room differences. This section also includes a technical assessment of eye-tracking vs. no eye-tracking groups (research question 3), as the results of this assessment were important for treatment of the other data. (2) We then consider a further breakdown between viewers reporting generally positive/negative experiences and tie these responses to reported emotion and looking patterns.

All participants completed all aspects of the study. Inspection of the histograms and Q–Q plots for the emotion and artwork appraisal items suggested, in general, approximately normal distributions, however, with some having a rightward or leftward skew due to the unidirectional nature of some scales. No outliers were detected (based on boxplots). Given our sample size and scale-based data, as well as the expectation of primarily using Analysis of Variance assessments which are robust to minor deviations, statistical tests for normality of distributions (e.g., Shapiro–Wilk) were not conducted (see Altman and Bland, 1995; Field, 2009). Assessment of homogeneity of variance for univariate comparisons are noted at the relevant locations below. In cases of violation of sphericity (repeated measures ANOVAs), Greenhouse-Geisser corrections were applied. Viewing time and eye-tracking data showed no violations in normality of distributions and variance homogeneity.

Comparison of participants wearing the eye-tracking glasses vs. those without glasses also did not reveal notable differences. This was assessed via repeated measures MANOVAs (Room as within subject factor, eye-tracking (Y/N) as between subject factor) considering either the nine artwork evaluation scales or the 20 emotion terms. Results showed no significant main effect of wearing glasses (Evaluation *F*(9,41) = 1.20, *p* = 0.32; Emotion *F*(20,25) = 1.07, *p* = 0.43), and no significant Room × Glasses interaction [Evaluation *F*(9,41) = 2.04, *p* = 0.06; Emotion *F*(20,25) = 0.72, *p* = 0.78]. Univariate tests for each individual scale also revealed no differences between Glasses conditions (Levene’s test for homogeneity of variances for all items *ns*). A repeated measures ANOVA for time spent viewing also showed no main effect of wearing eye-tracking glasses [*F*(1,45) = 0.19, *p* = 0.66)] or a Room × Eye–tracking interaction [*F*(1,45) = 0.55, *p* = 0.46)]. Therefore, all participants were combined in the following analyses.

### General Behavioral Responses

**FIGURE 3:**
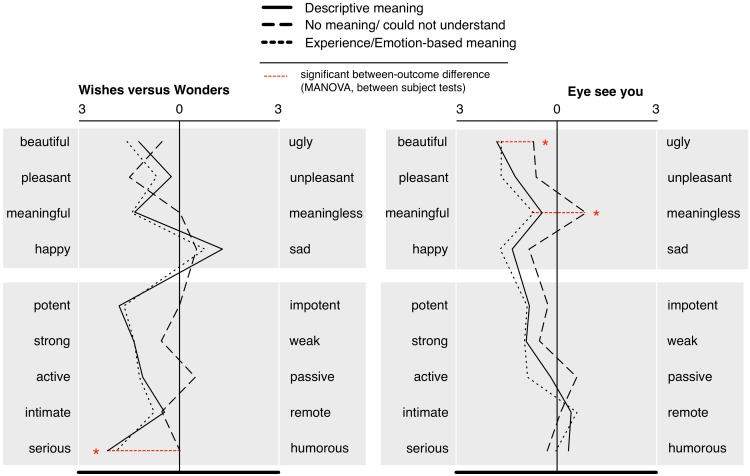
Differences in appraisals (post-viewing) between different types of answers to “what was the meaning of the art?” for the two rooms of installation art. ^∗^ and dotted line indicate significance at *p* < 0.05 (see also legend above).

**FIGURE 4:**
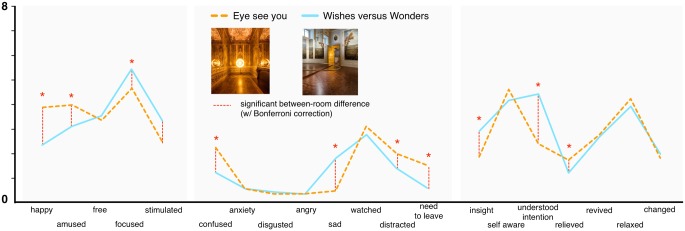
Reported emotions (post-viewing) in two rooms of installation art. All reported significant differences are based on univariate analyses with Bonferroni correction for multiple comparisons. ^∗^ and dotted line indicate significance at *p* < 0.05 (see also legend above).

The general responses to and appraisals of the artworks are shown in **Figures 2** and **3** (evaluations), **Figure 4** (reported emotions), and **Tables 1**, **2** (reported artwork meaning). Taken together, these data show notable differences in responses to the two installation works.

**Table 1:** What was the artwork meaning? Wishes vs. wonders.

Meaning type	Answer, translated from original German (participant number)
(31.4%) Experiential/emotion-based^a^	(3) To (the viewer) the pictures seem terrifying
	(5) It represents the cycle of life
	(8) I think it represents the connection between modern and classical art. The half ring is closed through the mirror and builds an unity in its reflection, also with the classical pictures on the opposite walls. Maybe it should represent a cycle that is linked to the acts of war that are shown in the pictures.
	(13) Modern art (optical illusion) linked to classical art. Transition. Infinity. Unity.
	(14) floating weightless sphere in space. No existence of space and time and gravity. The question of the persistence in life? Being exists only in the here and now? Reminds of the universe- > orbit of the sun.
	(16) It shows the human being as a part of the masses and it shows that its death is just one of many others and nothing special.
	(17) The artwork is astonishing. One feels drawn in to the situation by the mirror, like an untertow. It brings one down to earth again, how good our life in fact is, far away from battle, blood and death. But will this change in the future?
	(22) And again I don’t know, presumably: the history of the mankind is an eternal cycle. Again and again war and destruction.
	(30) Sadness, calm atmosphere
	(32) Cycle of war. The war cannot bring peace, but only new conflicts.
	(35) The blurring and interaction of the link between illusion and reality.
	(37) I think the intention was to show how the form alters depending on the angle from which the artwork is viewed. The circle/oval changes from each perspective
	(41) paintings of battles were shown and I think the meaning of the mirror was to lead the recipient to believe that everything was bigger and wider than depictable on the pictures. Although I found the circle in the middle was very impressive, because it seemed to float in the air, I could not think of any meaning
	(42) little, the optic circle reminded me a bit of the “doom”, the never-ending up and down of wars (with reference to the paintings)
	(43) In the battles many lose their lives and take the bullet for their country. Nature gets destroyed as well. General destruction. But also new, good things can emerge from that.
	(48) It plays at least with the optical illusion of a levitating object. It might advert to closed-ness or endlessness due to the imagery and the mirror.
(64.7%) Descriptive meaning	(1) detailed depiction of battles
	(2) moderate meaning in the art sector
	(6) Depiction of battles to provide documentation
	(7) The artworks are historical and show various battle scenes. They (the artworks) tell the visitors how and where battles were fought in the past and maybe they serve as a general reminder of the cruelties of war.
	(9) It shows war on several locations
	(10) Maybe the circle denotes a connection between the artworks (repetition of war)
	(11) To represent various battle scenes. How spatially extended they could be.
	(12) Several battles were depicted.
	(15) Mid-level meaning, because it depicts historical events, but without being a remarkably popular art piece.
	(18) paintings of various battles around the year 1700, historical, detailed
	(19) War
	(20) To me it was like an orbit
	(21) Seeing a piece of history
	(23) pictures of battles
	(24) The artworks were about battles at various places.
	(25) War theater, battle
	(26) Depiction of battlegrounds, tries to unify to a great artwork.
	(27) Depiction of a war scene. The pictures in a chronological order, beginning with gunfire etc.
	(28) the artist obviously had a preference for battles. I am impressed by the details of the paintings and I am wondering the whole time how long it took him to paint these artworks. Ha ha. It was surely exciting for the contemporary people to look at pictures of heroic battles. But I mostly could not figure out who was fighting whom.
	(29) A battle was depicted, that should be captured.
	(31) The presentation of various battles (historical moments), perhaps as achievements
	(33) It illustrates diverse battles of the 18th century.
	(34) Sundry presentations of battles.
	(36) I saw pictures of battles, these types of artworks have no meaning to me.
	(38) historical meaning (demonstration of power on one side) – uplifting-floating lightness on the other one
	(39) Depictions of several battles/wars, demonstration of power/efficacy of war –>Dead people, winnings, losses, changes etc.
	(40) battle fields, wars of the past
	(44) Depiction of historical battles.
	(46) Depiction of battles. Soldiers back then (in contrast to these days) were hailed as heroes, though, and thus their fall was dramatic. It looks rather heroic than war pictures nowadays, which meant to act as a deterrent.
	(47) painted war scenarios on several pictures, were imposing, but they all showed the same The artwork with the mirror (did not affect) me.
	(49) The paintings in the room depicted battles.
	(50) Battle, raid into a village, war
	(51) Showing several war-/battle strategies and depictions of “a whole”
(3.9%) No Meaning	(4) No idea
	(45) No idea


*categorization based on previous argument by Pelowski and Akiba (2011), Pelowski (2015). Assignment to categories done by three independent raters with disagreement decided by majority decision.*

**Table 2:** What was the artwork meaning? Eye see you.

Meaning type	Answer (participant number)
(35.3%) Experiential/emotion-based	(3) double meaning of life
	(5) To learn and understand other perspectives
	(6) magnificent ambience
	(7) The sort of “sun” bathes the room in an orange, very bright but pleasant light. Through the mirror one can see oneself everywhere a 100 fold. The meaning is very abstract and can certainly be interpreted in different ways. Maybe it wants to increase the self-confidence or just show that creating a pleasant and free feeling in such a small room is possible
	(8) I think it is supposed to show the limits of our visual perception and that we can still win by means of these new impressions. By playing with the complications and perceiving unfamiliar things, which are hard to interpret at first sight (like the other color perception)
	(11) Creating a world without color
	(14) seeing eye. Sees itself through all the mirrors. Insight question and recurring/continual. To understand oneself?
	(16) It resembles a sun and makes one feel more cheerful and more alert.
	(17) The angels on the ceiling look like they are going to put the flower wreath on one’s head. Impressive wall ornaments and one can see how impressive optical illusions have always been to people – the mirrors seem to reach ad infinitum. They carry one into a different world.
	(21) Humor. The artwork was very funny to me.
	(29) The warm light with the beautiful room composition made one happy
	(30) calming effect
	(34) Very garish light, it was hard to concentrate on the room, strongly distracted from the mirror image.
	(35) An extreme representation of width and infinity of spaces. Every apparent barrier (walls etc.) can bear additional space.
	(40) The influence of the yellow light on the experience of a joyful room.
	(42) strong light effect, oneself as “sinking” (head in the middle of the lamp with royal surroundings
	(46) One sees herself in a mirror with (awesome) light reflections that makes you look fancy. Congenial to the fancy room with the mirror. And in general congenial to the whole Winterpalais – apparently Mr Prince Eugen went for fancy things – and because it is still cool, a mirror was positioned. In this way we are all a bit prince/princess when we sparkle so nicely in our reflection.
	(47) Gorgeous ceiling painting with angels, it conveyed calmness, yellow light, that illuminated the room, that I found unpleasant, very beautiful ornament on doors and walls
(33.3%) Descriptive meaning	(1) reflection
	(2) dubious meaning of the positioned installation, rest of the room with very conspicuous beautiful adornments
	(13) Highlighting the room through light. Highlighting certain aspects of the room
	(18) splendid, historical
	(19) It is some Christian motive, but I do not know enough about this subject matter, to construe it in a right way.
	(20) It looks like one big eye, which can see everything because it lights the whole room. Perhaps a depiction of an “all-seeing eye”
	(23) golden room with angel
	(25) splendor, depiction of wealth, affluence
	(27) Abstract rebuilding of a sun
	(28) I felt watched by the angels with their dark eyes. Besides, in my opinion it shows that nobles in the past spent far too much money on art, just to boast. Apart from that I cannot say much about it.
	(31) I think it was just a well-lighted embellishment, that should demonstrate liking and pompous wealth, especially the flowers and tea cups/tankards cause wellbeing
	(38) a “beautified” picture of oneself (in the mirror)
	(39) Representation of sun, reflection in the mirrors
	(41) To me it looked like a living room with chimney and pleasant pictures of angels and flowers. The light had no meaning to me in this context and was incongruous.
	(43) Plenty of gold and the big lamp that spends “warmth”. The mirrors were very cool (similar to the hall of mirrors)
	(50) round reflecting sun
	(51) I associate the following with the artwork: warmth, sun, gold, beauty.
(31.4%) No Meaning	(4) no idea
	(9) ?
	(10) I cannot answer this question, to me it was not obvious. At least the light was pleasant.
	(12) No particular meaning, ornaments on wall and ceiling, bathed in yellow light
	(15) no big (meaning)
	(22) I don’t know. Maybe it was about highlighting something, drawing attention to something, to gain center stage?
	(24) the meaning is unknown
	(26) no idea
	(32) I don’t know
	(33) Unfortunately, I am not aware of the intention of the (art).
	(36) I looked at the room, to me it had no special meaning and I did not feel aroused/stimulated by the room
	(37) I don’t know
	(44) no idea
	(45) no idea
	(48) I heard how other visitors were talking about an “eye”. But I may not have noticed.
	(49) Unfortunately, I am not absolutely sure about the meaning. There was a big lamp positioned that dominated the room.


*categorization based on previous argument by Pelowski and Akiba (2011), Pelowski (2015). Assignment to categories done by three independent raters with disagreement decided by majority decision.*

#### Artwork Appraisals and Time Spent Viewing

Participants spent roughly twice as much time viewing *Wishes vs. wonders* (hereafter “*Wishes*”) (*M* = 4.24 min, *SD* = 3.17, min 1:00, max 20:10) than *Eye see you* (hereafter “*Eye*”) (*M* = 1.76, *SD* = 1.56, 0:43 to 10:52), with a *t*-test indicating that this difference was significant [*t*(46) = 7.75, *p* < 0.001]. This result may have partially been due to the larger dimensions of *Wishes*, but may have also been tied to the detailed murals on the three walls of the installation (see also related findings on artwork meaning, **Table 1**). We also found differences for appraisals. To analyze differences in the ratings, we conducted a repeated measures MANOVA with Room (2) as the within subject factor while including the nine evaluation scales. This revealed a significant main effect of Room [*F*(9,42) = 19.42, *p* < 0.001, *η_p_*^2^ = 0.81], and with individual univariate comparisons showing that, with the exception of “beautiful-ugly,” all scales differed significantly between rooms (all *ps* < 0.04, with Bonferroni adjustment for multiple comparisons). Generally, as shown in **Figure 3**, *Eye* was rated more highly on the hedonic measures (happy, pleasant), and was seen as more active, whereas *Wishes* was rated as more sad, but also more serious, meaningful, and generally more potent.

### Artwork Meaning

The above differences in evaluations could also be tied to viewers’ understanding of the meaning of the artworks. To analyze this, we first classified all short meaning answers into one of the three categories described in the Methods above: (1) direct or descriptive; (2) no meaning or assumed meaninglessness; (3) experience- or emotion-based descriptions. Meaning was classified, using the original German answers, by three independent raters trained in identifying the three levels. Raters showed a high level of initial agreement in their classifications, coming to the same classifications for >95% of answers; disagreements were settled by majority decision. All answers (English translations) are shown in **Table 1** (*Wishes*) and **Table 2** (*Eye*). Between rooms, there appeared to be notable differences in the use of the three types of meaning answers, with higher incidence of descriptive meanings in *Wishes* (64.7%), followed by experience-/emotion-based meaning (31.4%), and very low use of not understanding (4%). In contrast, *Eye* showed relatively less descriptive meaning (33.3%), higher experience-based meaning (35.3%) and higher lack of understanding (31.3%). A Chi square test (3 meaning types × 2 rooms) revealed these ratios to differ significantly between rooms [*χ*^2^(*N* = 102, 2) = 16.1, *p* < 0.001].

We also considered the likelihood that the same individuals would give the same type of meaning for both rooms despite the overall between-room differences. Individuals gave the same meaning type 49.0% of the time [well above a chance level of 33.3%; *X*^2^ (51, 1) = 5.7, *p* = 0.02], suggesting between-room within-participant consistency.

#### Meaning × Appraisals

The relation between the meaning answers and the artwork ratings (reported in **Figure 3**) was analyzed via two MANOVAs (meaning type set as between subjects factor) for each of the nine evaluation scales for each of the two rooms, conducted separately. No significant main effects were detected [*Wishes F*(18,82) = 1.02, *p* = 0.45, *η_p_*^2^ = 0.18; *Eye F*(18,82) = 1.15, *p* = 0.32, *η_p_*^2^ = 0.20]. Univariate tests for between-subjects effects for specific scales (with Bonferroni correction) revealed a significant difference in *Wishes* for “humorous-serious” [*F*(2,48) = 4.45, *p* = 0.02, *η_p_*^2^ = 0.16]. In *Eye*, differences were found for “beautiful-ugly” [*F*(2,48) = 3.16, *p* = 0.05, *η_p_*^2^ = 0.12) and “meaningful-meaningless” (*F*(2,48) = 5.95, *p* = 0.005, *η_p_*^2^ = 0.20; homogeneity of variances (Levene’s test) *ns*]. No significant differences were detected for time spent with the art (ANOVA, meaning category between subjects, conducted separately for each room).

#### Emotion

**Figure 4** displays the mean reported emotions experienced in the two rooms. Once again, to analyze between-room differences we conducted a repeated measures MANOVA with Room as the within-subject factor while including all 20 emotion scales. This resulted in a significant main effect of Room [*F*(20,26) = 3.87 *p* = 0.001, *η_p_*^2^ = 0.14]. Univariate comparisons between rooms for individual scales showing significant differences for happy, amused, and relief—all higher in *Eye*—and for feeling “focused,” “stimulated,” and “sad”—all higher for *Wishes.* Finally, looking to more cognition- or understanding-related scales, *Eye* had significantly higher reported feeling of distracted, confusion and “need to leave,” while *Wishes* had higher reported feeling of focused, insight, and “understanding the artist intention” (all *ps* < 0.05, following Bonferroni adjustment for multiple comparisons). Comparing the relation between emotion and artwork ratings and the two rooms, note also that the differences between the installations themselves would appear to explain much more of the artwork evaluations (81% as assessed via effect sizes) than the specific emotions experienced while viewing (14%).

#### Network Model of Emotion Terms

To investigate the emotional experience of viewers in more depth, we then used a Partial Correlation Network Model Analysis (Epskamp et al., 2012; Epskamp and Fried, 2016). This again assesses the partial correlations between items without an underlying assumption of higher-order factors. It can provide information regarding the centrality, interconnections, and specific importance of items in defining the global experience. Our main aim here was to investigate how the emotions related to each other, as assessed via their connections, as well as to consider between-room differences.

Analysis was conducted using the R package *qgraph* (Epskamp et al., 2012) with “graphical LASSO” regularization assessing the same 20 underlying emotions as above, conducted separately for each room. The hyperparameter (γ) was set to 0.2 for both rooms’ models. This determines the sparseness of the network (the number of connections), by eliminating spurious connections. Although literature is only now emerging for modeling procedures, previous publications have suggested a typical hyperparameter at around 0.25 for hypothesis-driven research (a higher total is more conservative; e.g., see Barber and Drton, 2015). However, Epskamp and Fried (2016) also suggest that researchers should make an informed decision to adjust the parameter, typically toward a less conservative number, based on their own study conditions. Therefore, due to the exploratory nature of the study, we determined that 0.2 gave an optimal balance between conservatism while still retaining some key connections between items.

**FIGURE 5:**
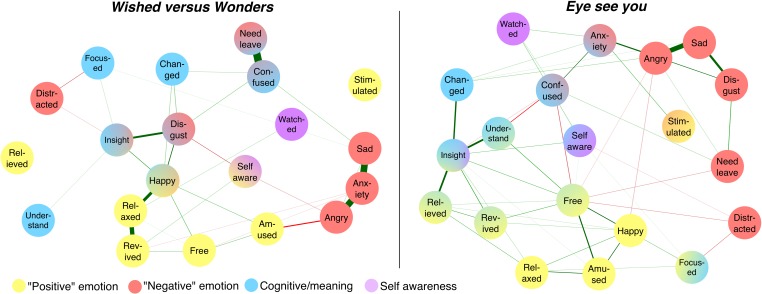
Network model of reported emotions in the two rooms of installation art. Analysis conducted using the R package *qgraph*, graphical LASSO regularization (Epskamp et al., 2012). Individual emotions shown as circles; green lines indicate positive partial correlations (surviving the regularization procedure); red lines indicate negative partial correlations. Line thickness indicates strength of correlations. Relative distance between items suggests strength of connection as a function of the entire network. The colors of the circles correspond to the basic expected nature of the emotion (negative, positive, cognitive/meaning-related, self-awareness). Colors assigned to optimize the presentation of the results.

**FIGURE 6:**
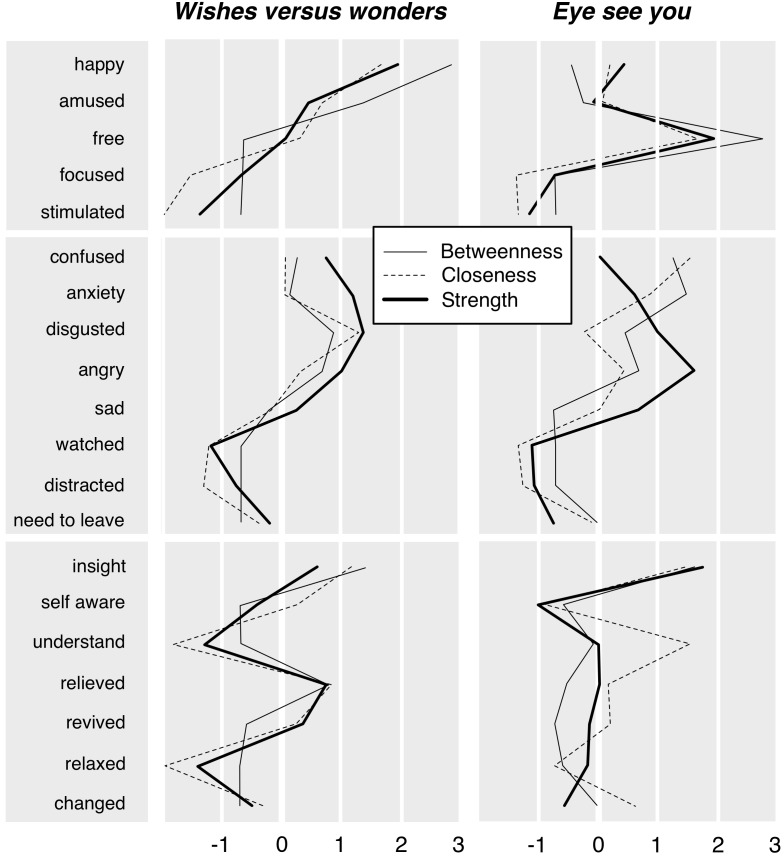
Closeness, betweenness, and relationship strength of emotion terms in the network model.

The final models are shown for the two rooms in **Figure 5**. The individual emotions are shown as circles; green lines connecting the circles indicate a notable positive partial correlation (surviving the conservative regularization procedure); red lines indicate a negative partial correlation. Thickness of lines indicates strength of correlations, with no line suggesting that a relation is minimal. The colors of the circles were assigned by ourselves and correspond to the basic expected nature of the emotion (negative, positive, cognitive/meaning-related, self-awareness). We also report the calculated “closeness” (relative centrality within the entire model, calculated as the inverse sum of shortest distances from all other emotion nodes to one focal node), “betweenness” (degree to which a node is on the shortest path between other nodes), and “strength” (sum of the weights of connections that a node has) for all emotion terms in **Figure 6**. The centrality measure of closeness also represents the inverse sum of shortest distances from all other nodes (emotions) to one focal node.

The main result appeared to be that generally more positive emotions (yellow circles) and negative emotions (red circles) were located on opposite sides of the network with a general negative correlation between these items (red lines), suggesting a low likelihood of reporting both sets of emotions in one experience (see also the generally low “Closeness” measures, **Figure 6**, for both emotion types). At the same time, mediating these groups were more cognitive responses (blue circles)—confusion, insight, and especially self-awareness. In both artwork cases, in order to move between a negative and a more positive responses (tracing a pattern along green connecting lines), required a movement through self-awareness. Self-awareness, in turn, fed into “understanding intention,” relief, and insight. This might especially be connected to the path toward an experience-based meaning evaluation as seen in *Eye*. The individual connections among the emotions can also be taken into account. In addition to line thickness and red/green color, the significance of such connections within the overall model is also evidenced by the measure of “Strength.” As can be seen in the **Figures 5** and **6** in *Wishes* disgust, insight and happy are strongly connected. This suggests that people may initially feel disgusted by the artwork but then as they gain insight feel happy. Since happy is then strongly connected to relaxed (and relaxed to revived), the feeling of happiness may then persist and lead the person to feel relaxed and revived. Though the art experience may have started off negatively with disgust, as people gained insight they may have felt like they gained some understanding (insight) and had an overall positive experience.

In *Eye*, we do not find the above pattern. Even though Insight and revived are still connected, this connection does not run through happy or relaxed. In this case, insight mainly connects to feeling changed and feeling a sense of understanding of the art. What may be happening with *Eye* is that people gain insight into the art which makes them feel they understand it, as well as that they feel changed by it, and finally that they feel a sense of relief. The experience seems therefore more “cognitive” with a steady pattern of understanding the work than in *Wishes* where insight seems to work more as a pathway to go from feeling bad (disgusted) to feeling good (happy, relaxed, and revived). However, in both *Wishes* and *Eye*, sad, anxiety, and angry are also highly connected to each other but minimally connected to other nodes. Thus, if a viewer feels one of these three emotions, they are likely to feel all three during their experience, but are unlikely to report feeling anything else.

#### Emotion × Meaning

We also assessed the potential differences in reported emotion between the three meaning types (MANOVAs conducted for each of the 20 scales × the two rooms, separately). Analysis again revealed no significant main effects for both rooms. Additionally, for *Wishes*, no notable differences in univariate tests of between-subjects effects within specific emotions were detected. For *Eye*, tests for between-subjects effects revealed one significant difference (with Bonferroni correction, alpha = 0.0025) regarding “having understood the artist intention” [*F*(2,48) = 9.18, *p* < 0.001], which was highest with descriptive meanings, slightly lower for experience based, and lowest for no meaning. Without Bonferroni correction, differences were also detected for feeling “changed” [*F*(2,48) = 5.92, *p* = 0.005], and “insight” [*F*(2,48) = 3.97, *p* = 0.02]—which were generally higher for an experience-based meaning. Differences were also found for “confused” [*F*(2,48) = 5.91, *p* = 0.005], higher for individuals giving “no meaning” answers (tests for homogeneity of variance for univariate comparisons, *ns*).

### Eye-Tracking

#### Fixations and Fixation Duration

BeGaze software (version 3.5.101) was used for semantic gaze mapping. This allows frame-by-frame assessment and manual coding of fixation counts and fixation durations as they relate to researcher-defined regions of interest (ROI) within the recorded video. For the purpose of our analysis, and in order to give a general overview of where participants were looking in each room, we identified three main ROIs for each room. These included: (1) the artworks (defined as any component that had been installed by the artist); (2) the ambient environment (including walls, ceiling and ornamentation); and (3) the self (either viewed directly or as a reflected image).

Descriptive statistics for both rooms—with fixation counts, raw fixation times, fixation durations, and percentage of looking time as they relate to viewing either walls/ceiling, self, or artwork—are displayed in **Table 3**. Participants tended to look at the *Wishes* installation as well as all of its components longer (fixation time) than with *Eye*. They similarly showed more fixations. Following typical eye-tracking studies, these aspects were also themselves highly correlated (total fixations and total fixation time, *Wishes*, *r* = 0.981; *Eye*, *r* = 0.968). The individual fixation durations for *Eye* tended to be longer, although paired sample *t* tests for duration of looking for the different elements showed no significant differences.

**Table 3:** Eye-tracking results.

	Fixation time (ms)		Fixation count		Fixation duration (ms)
			
	Mean (SD)	% Total	Mean (SD)	% total	
*Wishes vs. wonders*					
Artwork	27,299.6 (30,960.2)	34.5% (36.0)	104.8 (106.7)	25.4% (29.1)	221.5
Walls/ceiling	108,459.2 (115,086.3)	66.3% (35.9)	471.9 (464.5)	73.1% (29.9)	204.1
Self	3,071.6 (3,247.2)	4.4% (5.8)	10.7 (10.8)	2.2% (1.9)	295.6
Total	131,781.0 (108,643.9)		583.8 (431.2)		212.8
*Eye see you*					
Artwork	8155.9 (15,003.7)	11.6 (12.14)	28.5 (42.2)	11.5% (12.0)	225.3
Walls/ceiling	48,869.5 (44,718.4)	83.8% (13.4)	208.0 (172.7)	85.7% (12.9)	225.0
Self	3,303.8 (2,946.8)	7.0% (6.1)	5.4 (6.8)	2.8% (3.2)	378.4
Total	59,227.9 (54,724.7)		241.9 (189.2)		235.5


When considering the percentage of total looking time/fixations devoted to artwork, self, or room elements (wall/ceiling), participants also tended to look at the artwork in *Wishes* (defined as both the room-length mirror and/or the embedded ring) roughly three times more (34.5%) than in *Eye* (11.5%). The majority of other looking duration/fixations were devoted to the environment, followed by roughly 5% of time spent looking at the self. Univariate t-test comparisons for percentage of duration/fixations devoted to wall, self, or art (with Bonferroni correction, adjusted alpha = 0.0084, based on six comparisons) showed significant between-room differences for fixation time with the artwork [*t*(17) = 3.50, *p* = 0.003] and the wall [*t*(16) = 3.02, *p* = 0.008]. Fixation duration on self and all comparisons of fixation number comparisons were *ns*.

#### Eye-Tracking Results × Appraisals, Emotion, and Meaning

Assessment of correlations between fixation durations on the artworks, self, or room and the viewers’ appraisals (shown in **Table 4**) did not show any significant relations for either room following Bonferroni correction. Without correction, trends were detected for *Wishes* regarding fixation duration on the artwork and “happy-sad” and “seriousness-humorousness.” Here, more focus on the art, and presumably not on the battlefields, resulted in a happier but less serious appraisal. This latter assessment of seriousness was also reflected in the percentage of attention given to either art or room (see **Table 4**). A notable correlation was also found between fixation duration on the room and feeling the art to be more meaningful. Moreover, focusing more on the self correlated to assessing the art as more distant. No significant/notable patterns were uncovered for *Eye*. For emotion as well, no significant correlations were detected for either room following correction for multiple comparisons.

**Table 4:** Correlation of artwork ratings and percentage of fixation time on artworks.

	Fixation time			Percent of fixations		
		
	Artwork	Wall	Self	Artwork	Wall	Self
*Wishes* vs. *wonders*						
Beautiful: ugly	-0.271	-0.264	0.212	0.225	-0.176	0.407
Active: passive	0.129	-0.377	-0.289	0.136	-0.106	-0.072
Pleasant: unpleasant	-0.226	0.258	0.368	-0.202	0.178	0.167
Happy: sad	-0.488^∗^	0.359	0.271	-0.416	0.419	0.171
Strong: weak	-0.119	-0.242	-0.487	0.170	-0.094	-0.056
Intimate: distant	0.153	-0.012	0.717^∗^	-0.131	0.080	0.309
Meaningful: meaningless	0.186	-0.508^∗^	-0.329	0.457	-0.409	0.102
Serious: humorous	0.598^∗^	-0.390	-0.194	0.634^∗^	-0.574^∗^	0.345
Potent: impotent	0.177	-0.319	-0.130	0.343	-0.262	0.229
*Eye See you*						
Beautiful: ugly	0.012	0.113	0.107	0.102	-0.136	-0.024
Active: passive	-0.284	0.153	0.008	-0.288	0.196	0.333
Pleasant: unpleasant	-0.045	0.141	0.127	-0.019	0.008	0.181
Happy: sad	0.133	-0.158	0.265	0.144	-0.272	0.309
Strong: weak	-0.267	0.208	-0.035	-0.320	0.323	0.068
Intimate: distant	0.231	-0.057	-0.031	0.443*	-0.373	-0.336
meaningful: meaningless	-0.100	0.169	-0.144	0.019	-0.034	-0.018
Serious: humorous	0.167	0.279	0.140	0.257	-0.296	0.028
Potent: impotent	-0.065	0.219	0.015	-0.069	0.027	0.025


*Negative correlation implies relation to leftmost term on bipolar scale. Positive correlation implies relation to right term. ^∗^Indicates significance at *p* ≤ 0.05, uncorrected. No correlations maintained significance following Bonferroni correction for multiple comparisons (familywise corrected alpha = 0.0028).*

An analysis of the relationship between the eye-tracking results and the meaning categories (ANOVAs for meaning type, conducted separately for the six fixation durations and percent of fixation duration assessments described above) did not lead to significant or notable findings. Note that the eye-tracking data was only conducted on half of the participants, leading to a quite small sample, which may not have detected more subtle relationships.

### Positive Vs. Negative Experiences and Related Factors

**FIGURE 7:**
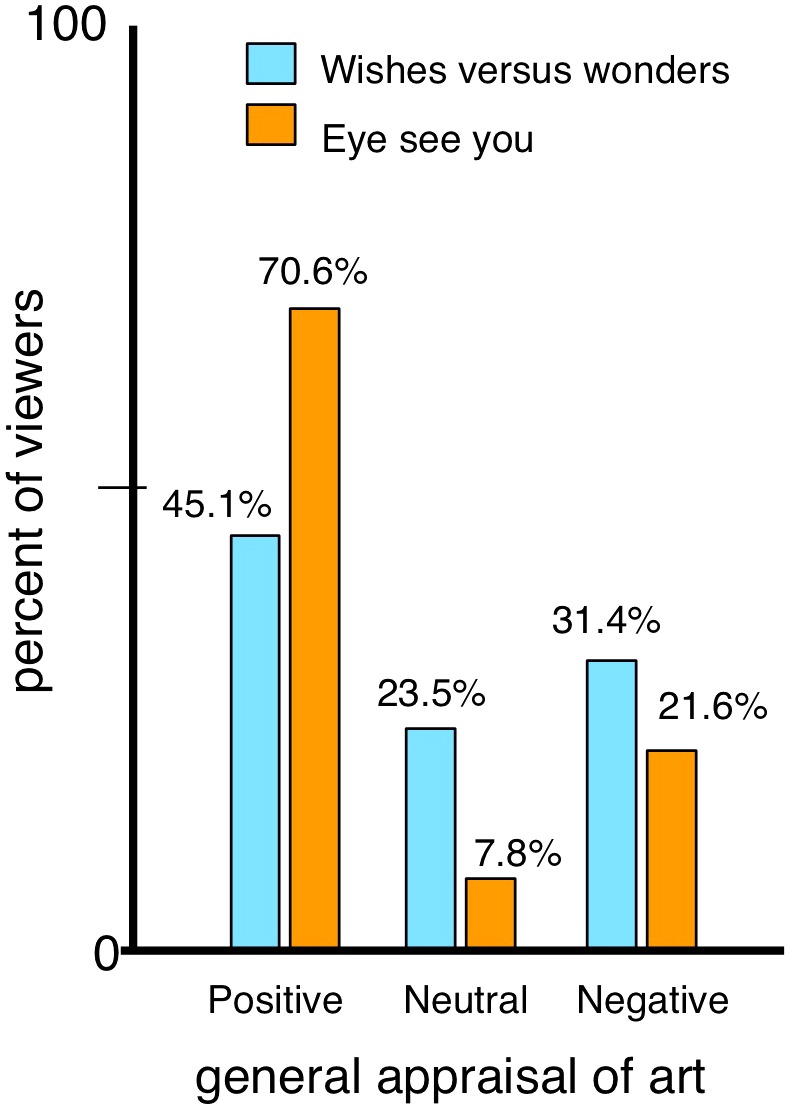
Distribution of generally positive, neutral, and negative appraisals of artworks. Based on answers to 7-point scale (the art was pleasing-displeasing).

To assess the factors positively influencing the viewers’ experiences in more depth, we compared individuals who reported a positive vs. a negative experience in the two rooms. This was done by considering answers to the artwork evaluation “pleasing-displeasing,” considered as a general assessment of preference or enjoyment of the art (Silvia, 2005b). A breakdown of responses (separated between generally “pleasing,” score of “1–3”; “displeasing,” “5–7”; and “neutral,” score of “4” on 7-point scale) are shown for both rooms in **Figure 7**. *Eye* had more individuals giving a generally positive rating (70.6%) vs. *Wishes* (45.1%). *Wishes* in turn had more negative (31.4%) and neutral (23.5%) responses as compared to *Eye* (pleasing = 21.6%; neutral = 7.8%). This also suggested less ambivalence, as surmised from neutral scores, with *Eye*.

**Table 5** shows the correlation between the “pleasing-displeasing” and the other artwork ratings. As expected, the ratings were generally highly correlated with other hedonic measures such as happiness and beauty. There was also general negative correlation with scales tied to dynamism (potency, activity), and especially “serious-humorous” (although not significant after Bonferroni correction). The same general results were also found when participants were broken into the three groups of those giving a positive, neutral or negative answer to liking the art. These were assessed with a set of two MANOVAs (pleasing-neutral-displeasing Appraisal as between subjects factor) conducted with all eight remaining scales for each room, separately. These showed a significant main effect of Appraisal in both rooms [*Wishes*, *F*(Pillai’s Trace, 16,84) = 2.58, *p* = 0.003, *η_p_*^2^ = 0.33; *Eye*, *F*(Pillai’s Trace, 16,84) = 1.82, *p* = 0.04, *η_p_*^2^ = 0.26]. Tests of between subject effects for each room revealed significant differences in *Wishes* for beautiful, happy, and humorous (higher for good vs. the other appraisals, all *p*s < 0.03, corrected for multiple comparisons). Similarly, for *Eye*, differences were detected for more “beautiful” and more “happy,” as well as for “strong-weak” and “intimate-distant,” with the negative appraisal coinciding relatively more with the leftward terms on the scale (all *ps* < 0.05, corrected).

**Table 5:** Correlation between “pleasing-displeasing” artwork assessment and other art ratings.

	*Wishes vs. wonders*	*Eye See you*
Beautiful: ugly	0.398	0.601^∗^
Active: passive	-0.286	0.198
Happy: sad	0.602^∗^	0.629^∗^
Strong: weak	-0.272	0.251
Intimate: distant	-0.302	-0.366
Meaningful: meaningless	-0.049	0.283
Serious: humorous	-0.328	-0.241
Potent: impotent	-0.077	0.149


*^∗^Significant correlation following Bonferroni correction for multiple comparisons (familywise). Adjusted alpha = 0.0031 for 16 comparisons.*

**FIGURE 8:**
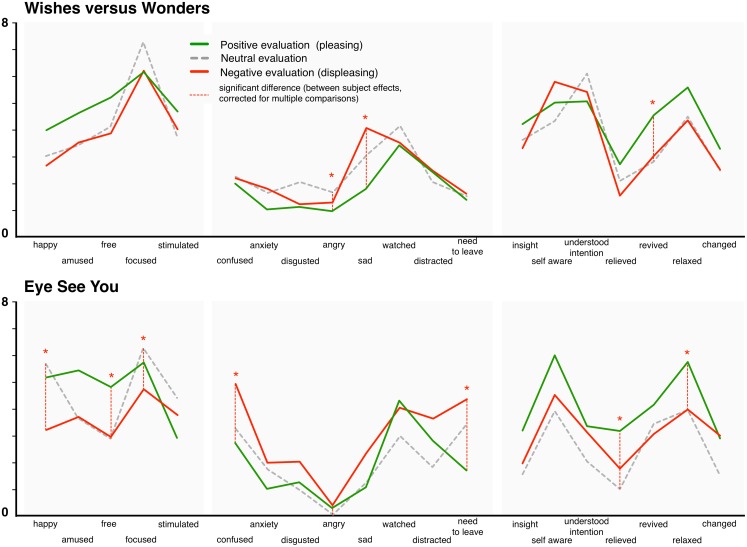
Comparison of Mean reported emotions among individuals giving positive, neutral, and negative general appraisals of artworks. ^∗^ and dotted line indicate significance at *p* < 0.05 (see also legend above).

For emotion (see also **Figure 8**), a similar set of two MANOVAs were conducted for all 20 scales in each room, separately. Once again, these revealed a significant main effect in each room [*Wishes*, *F*(Pillai’s Trace, 40,58) = 1.76, *p* = 0.025, *η_p_*^2^ = 0.55; *Eye*, *F*(Pillai’s Trace, 40,52) = 1.79, *p* = 0.025, *η_p_*^2^ = 0.58]. In *Wishes*, individual tests of between subject effects showed differences for sad, angry (both higher for negative evaluation compared to positive evaluation), and revived (higher for positive, all *ps* < 0.025, corrected). In *Eye*, differences were detected again for angry (higher for negative), happy (higher for positive), as well as for confused and need to leave (higher for negative), for relieved, relaxed, and free (higher for positive), and for focused (highest for neutral but also relatively high for positive; all *ps* < 0.05, corrected). Interestingly, in both rooms it appeared that those ending with a neutral art rating tended to show the highest focus, possibly due to the need to deal with an ambivalent semantic or experiential situation.

Interestingly, when we looked to the general pleasing/displeasing artwork appraisals and assessed artwork meaning, we did not find a notable pattern. Rather, as shown in **Table 6**, in both bad and good appraisal cases, individuals tended to rely most heavily on descriptive meaning. Contrary to our working hypothesis, emotion-based meaning did not appear to tie to more positive assessments in either installation. Further, giving an answer that the art had no meaning or that an individual could not understand the art also did not appear to necessarily lead to negative assessments. This was especially true for *Wishes*, where no individuals giving a “no meaning” response coincided with a displeasing assessment (*X*^2^ for meaning types × outcome type for both rooms: *ns*).

**Table 6:** Distribution of types of answers to “What did the artwork mean?” when viewers appraise art as generally good, neutral, or bad.

	% Meaning type usage for good, bad, neutral artwork appraisals		
	
	Descriptive meaning (%)	No meaning (%)	Emotion-/experience-based meaning (%)
*Wishes vs. wonders*			
“good” appraisal	56.5	8.7	34.8
Neutral appraisal	75.0	0.0	25.0
“bad” appraisal	68.8	0.0	31.3
*Eye see you*			
“good” appraisal	33.3	27.8	38.9
Neutral appraisal	50.0	25.0	25.0
“bad” appraisal	27.3	45.5	27.3


Time spent in the rooms and eye-tracking data (raw fixation times and percent of time spent looking at artwork, room, or self) also showed few significant or notable differences. When breaking down the ratings into the three groups of those giving a good, neutral, or bad rating, only one result showed significance (with or without correction). Fixation time on the room with *Eye* [*F*(2,18) = 8.79, *p* = 0.002] was higher among those giving a bad appraisal of the art. Neutral and good appraisals showed roughly the same looking time.

## Discussion and Conclusion

This study considered, in an exploratory fashion, the experience of installation art encountered in the museum. We investigated whether this unique art medium—which has become increasingly common yet is often difficult for both the interested researcher and for the novice viewer to assess or appreciate—can be meaningfully evaluated in terms of the emotional and semantic experience. We further considered how experiences may differ between specific artworks or in relation to viewers’ positive/negative evaluations, as well as more technical questions related to how individuals approach the spaces, namely, as an integration of room and installation or as stand-alone pieces. Finally, we evaluated the efficacy of our self-report approach, collection and organization of reported emotions, as well as the use of eye tracking.

Regarding our first research question: Do the two artwork installations produce distinct emotional or evaluative responses that may differ within-subjects, and could these be captured? We found that indeed the two installation examples produced distinct emotional and evaluative responses. Participants, even when viewing both rooms in a counterbalanced order, tended to have a more positive and emotional experience with *Eye see you*, with them reporting significantly higher happiness, feeling of pleasantness, and active nature of the art. In contrast, for *Wishes vs. wonders*, participants reported more sadness, less pleasantness, but also more meaningful, potent, intimate and serious encounters, as well as more feeling of understanding the intention of the artist. These differences appeared to be due to the specific installation elements in conjunction with the pre-existing room features, as well as at least partially the artist intention. In *Eye*, the warm light cast by the artificial sun tended to drive the interpretations and emotions, whereas in *Wishes*, the paintings tended to lead to a focus on war and death that were tied to the presence of battlefield depictions. These were part of the pre-existing environment, but further highlighted by the artist’s intervention.

These differences could themselves suggest two different responses to the art—that is, between a more “emotional/harmonious” and a more “cognitive/intellectual” engagement. Such a dichotomy in potentially positive but qualitatively different reactions has been recently posited in models of art processing (Pelowski et al., 2017b). This suggestion is also supported by the emotion network model, which shows a more central position for basic positive emotions such as happiness in *Eye see you* and a more central position for cognitive responses—insight, feeling changed, confusion—in *Wishes vs. wonders*. This is also suggested by the meaning interpretations. Visitors to *Wishes* tended to focus much more on the semantic level. Almost every participant in the descriptive category tied the meaning to the battle scenes. This result would also partially explain the above differences in seriousness and sadness attributed to the art.

The focus on room and art elements also suggests an answer to our sub-question of whether viewers would appreciate the installed elements as stand-alone artworks or as components that interact with, and may play off of, the ambient environment. Viewers did appear to specifically consider the interplay of these factors when making evaluations or as part of their perceptual/emotional experience. In both cases (and especially with *Wishes*) viewers tended to interpret the artist-installed portions in light of the ambient environment, arriving at a final interpretation that more or less successfully combined both elements. This suggests that—even if this approach may be a relatively newer way of engaging art made necessary with installation and other contemporary examples—individuals with even limited art knowledge seem capable of this response. Such interactions can also be empirically captured. Viewer interpretations of the installations also tended to align with existing art critical and curatorial discussions, showing that lay viewers could indeed pick up on and appreciate the often ambiguous or esoteric nature of the installations.

Especially in *Wishes vs. wonders*, it could also be suggested that the rather traditional, semantic content facilitated interpretation—thus, the very low incidence of not understanding. Note, however, for those who mentioned the mirror and ring, this was usually in the context of not truly grasping their purpose or trying to connect these to the endless cycle of war or the human condition. Those suggesting an experience- or emotion-based interpretation in turn suggested sadness, terror, calm, as well as infinite or forced questioning of perception or life meaning. In *Eye*, participants providing descriptive answers focused primarily on a “sun,” or provided a recap of the installation elements and/or the ornate walls and ceiling. Those focusing on experience/emotion noted feelings of warmth, alertness, joy, but also (presumably because of the mirror and bright light) forced consideration of vision. (No viewer mentioned melancholy, which had been suggested as a potential reaction by the artist). The higher incidence of “no meaning” answers presumably tied to this room’s lack of more obvious mimetic elements. However, when looking at those individuals who had a generally positive vs. negative art experience, we did not find that the respective meaning interpretations—dividing between descriptive and more emotion-/experience-based answers—showed significant distribution differences. This in itself is quite interesting, as it tends to work against the argument that such latter interpretations are the “proper” way to enjoy installation art. It also is contrary to our own past findings in museum studies with abstract art (Pelowski, 2015) that meaning interpretations which focus on the experience rather than a recount of features or descriptive aspects tended to correlate with higher enjoyment and engagement.

The lack of connection between meaning assessments and ratings could be due to our use of lay viewers who often base their aesthetic judgment on emotionality (Leder et al., 2012, 2014). It could also be that the focus on the content rather than emotion for many viewers related to more overt (if largely subconscious) strategies used by participants to, for example, distance themselves from the emotion related to these depictions by basing their interpretation toward descriptions. Similar results have been found by Kemp and Cupchik (2007); see also Cupchik (2013) who presented viewers with a range of positive and negative paintings and found that viewers who wanted to see the paintings with negative themes a second time did so primarily because they evoked thoughts rather than feelings. Whereas in *Eye*, where there was both less readily graspable semantic content and generally more positive emotions, viewers may have been more likely to focus on the ambiguity and/or their emotional experience. It may also be that our sample did not detect a tie to meaning due to the limited size.

At the same time, although not detected in the quantification of written meaning answers, the patterns found in the network models of emotion, especially for *Eye*, did tend to suggest at least some evidence for general evolutions or importance of reflection and insight. Again, in the relationship between emotion reports we found that positive and negative emotions tended to be separate, however, with the possibility for a movement between these responses mediated by confusion and especially self-awareness, which also fed into “understanding intention,” relief, and insight. Such a progression has again been specifically suggested in models of transformative art (Pelowski and Akiba, 2011). Such interactions between specific emotions are obvious key candidates for future more in-depth research. Studies could especially attempt to identify which artwork aspects might trigger which emotions or interpretations within a flow of experience. This also raises the potential importance of network models in study with art.

The emotion results, even in their present level of complexity, did on the other hand support our second research question regarding ability to divide reactions to the rooms into generally positive or negative experiences. While the approach to meaning was not a major driver of appraisal, nor was understanding, we did find that the general valence of emotions experienced showed the most important tie to the general positive/negative evaluation. In both rooms, those who evaluated the art as happy tended to have a positive viewing experience, whereas those reporting sadness had generally negative evaluations. Although this finding is more facile than we had expected, it may be an important point of departure for future research. Certainly, previous studies have found similar tie between positive/negative emotional valence and appraisals of art. Such a focus on positive feelings might be a useful candidate for setting up future museum programs or art education. It is also important to note that positive and negative emotions might also occur at the same time (e.g., Hosoya et al., 2017). These aspects, especially due to their emphasis on induced emotions, would also be a key candidate for future installation art research, and might be explored by, for example, network models in tandem with meaning analysis.

Finally, we also further supported the use of mobile eye tracking for museum studies, as there was no evidence that such use changed the emotional experience or appraisals of the viewers. This is in keeping with other real-world art viewing studies which also showed similar results (Mitschke et al., 2017). At the same time, although looking patterns did differ between artworks, they did not appear, at least at the level of our present assessment, to play a notable role in differentiating the nature of the outcomes such as other emotions, types of meaning, or generally positive/negative appraisals. This also is in line with previous study by Gartus et al. (2015), which also considered art in conjunction with background context (gallery space vs. street backdrops) and which found that longer artwork viewing durations did not necessarily tie to more positive evaluations. Note again, however, that our sample was split in half for the eye-tracking manipulation (for experimental control). Thus, we may not have had sufficient power to detect subtle differences, and this topic remains a largely under-explored avenue for future empirical work.

This study also has other caveats and questions for future research. Future analyses should include larger samples, a wider range of art, and more focused hypotheses. Note also that we included a handful of particularly salient items. There are a wealth of other factors—more specific movement, body responses, other senses, that might and should be considered. Future study may also assess which of the above findings can generalize to all or most installation art or *in situ* art study examples. That said, we would argue for some key implications and conclusions that can be drawn from this research. This includes the ability to meaningfully assess people’s experiences of installation art and to uncover art/person differences. It is clear that many viewers can and do have meaningful reactions to installation art, driven largely by their generally positive or negative emotions, rather than where they look or their interpretations of meaning. We also thus support the sensitivity or efficacy of the included measures, laying out a paradigm that might be employed in future research. We also show that researchers should further examine the experience of installation artworks as they provide optimal contexts for more ecologically valid investigations of the experience of art.

## Author Contributions

MP, HL, VM, EV, TB, and AH-A contributed to the study design and the execution of the research study. MP, VM, GG, ES, EV, and TB contributed to the data analysis. MP, HL, VM, GG, ES, and PT contributed to the writing of the paper.

## Conflict of Interest Statement

The authors declare that the research was conducted in the absence of any commercial or financial relationships that could be construed as a potential conflict of interest.

## Footnotes

1 Held in the Winter Palace of Prince Eugene of Savoy, Vienna; officially a cooperation between Belvedere, Vienna, Thyssen-Bornemsiza Art Contemporary Collection, Vienna, and The Juan and Patricia Vergez Collection, Buenos Aires.

2 https://www.yatzer.com/olafur-eliasson-baroque-baroque

3 Note that this study also employed a number of other questionnaires (available upon request). These were used for a separate assessment of museum interaction conducted by a collaboration partner, administered last after the above reported procedure and questions and will not be referred to in our analyses.

4 We also recorded total fixations and total fixation duration. However, these measures are typically highly correlated. It is in turn suggested that duration may be a clearer measure of attentional focus.
